# Changes in Psychological Distress During the COVID-19 Pandemic in Japan: A Longitudinal Study

**DOI:** 10.2188/jea.JE20200271

**Published:** 2020-11-05

**Authors:** Hiroyuki Kikuchi, Masaki Machida, Itaru Nakamura, Reiko Saito, Yuko Odagiri, Takako Kojima, Hidehiro Watanabe, Keisuke Fukui, Shigeru Inoue

**Affiliations:** 1 Department of Preventive Medicine and Public Health, Tokyo Medical University, Tokyo, Japan; 2 Department of Infection Prevention and Control, Tokyo Medical University Hospital, Tokyo, Japan; 3 Division of International Health (Public Health), Graduate School of Medical and Dental Sciences, Niigata University, Niigata, Japan; 4 Department of International Medical Communications, Tokyo Medical University, Tokyo, Japan; 5 Graduate School of Advanced Science and Engineering, Hiroshima University, Hiroshima, Japan

**Keywords:** K6, novel coronavirus, mental health, general population

## Abstract

**Background:**

This longitudinal study aimed to examine the changes in psychological distress of the general public from the early to community-transmission phases of the COVID-19 pandemic and to investigate the factors related to these changes.

**Methods:**

An internet-based survey of 2,400 Japanese people was conducted in two phases: early phase (baseline survey: February 25–27, 2020) and community-transmission phase (follow-up survey: April 1–6, 2020). The presence of severe psychological distress (SPD) was measured using the Kessler’s Six-scale Psychological Distress Scale. The difference of SPD percentages between the two phases was examined. Mixed-effects ordinal logistic regression analysis was performed to assess the factors associated with the change of SPD status between the two phases.

**Results:**

Surveys for both phases had 2,078 valid respondents (49.3% men; average age, 50.3 years). In the two surveys, individuals with SPD were 9.3% and 11.3%, respectively, demonstrating a significant increase between the two phases (*P* = 0.005). Significantly higher likelihood to develop SPD were observed among those in lower (ie, 18,600–37,200 United States dollars [USD], odds ratio [OR] 1.95; 95% confidence interval [CI], 1.10–3.46) and the lowest income category (ie, <18,600 USD, OR 2.12; 95% CI, 1.16–3.86). Furthermore, those with respiratory diseases were more likely to develop SPD (OR 2.56; 95% CI, 1.51–4.34).

**Conclusions:**

From the early to community-transmission phases of COVID-19, psychological distress increased among the Japanese. Recommendations include implementing mental health measures together with protective measures against COVID-19 infection, prioritizing low-income people and those with underlying diseases.

## INTRODUCTION

The novel coronavirus infection that started in Wuhan, China, has spread throughout the world, and the World Health Organization (WHO) has officially declared COVID-19 a pandemic. As of June 15, 2020, the total number of infected individuals has exceeded 7 million, while the number of deaths has reached 400,000.^1^ The rapid spread of COVID-19 has created fear and anxiety about contracting the virus.^2^^,^^3^ It has also caused a lack of access to medical care and restrictions in daily life, in addition to having a major economic impact due to the suspension of businesses and unemployment.^4^^–^^6^ It has been noted that these circumstances might not only affect physical health but also lead to the deterioration of mental health, which in turn, would make the implementation of preventive actions, such as refraining from going out and social distancing, more challenging. We believe that this will result in a vicious cycle that will ultimately lead to the spread of the infection. In addition to maintaining the mental wellbeing of individuals, mental health measures are important for early suppression of transmission of the infection.^3^^,^^7^

To consider the need for mental health measures during the COVID-19 pandemic, it is necessary to first formulate an epidemiological description of the severity of related mental health problems. According to the results of a recent cross-sectional survey in China, 16–28% of citizens reported anxiety and depression.^8^ However, since other cross-sectional studies were unable to compare these data with pre-COVID-19 levels, the magnitude of its impact is unknown. To clarify the pandemic’s impact on the general public’s mental health, a longitudinal study that would allow tracking of subjects from the early phase before the pandemic is necessary.^9^ A study by Wang et al included a survey at two time points to determine the impact of mental health; however, their study recruited different individuals in each of the two surveys, so they could not assess inter-personal changes in psychological distress.^10^ To the best of our knowledge, no longitudinal studies have yet investigated the same individuals at two time points.

Furthermore, when devising mental health actions, it is necessary to identify the specific characteristics of individuals who are at a higher risk. It has been pointed out that older people with underlying diseases, those with mental health problems before the pandemic, children, and medical workers might be at high risk for the deterioration of mental health.^5^ In China, gender, age, and educational background were cited as relevant factors.^8^^,^^11^^,^^12^ Since only cross-sectional studies have been conducted, it is difficult to determine whether individuals’ mental health deteriorated after the onset of the COVID-19 pandemic, or whether their mental health was poor prior to the pandemic.

With this in mind, the purpose of this study was to: i) investigate the degree of change in mental health at the population level, and ii) identify the high-risk groups prone to mental health deterioration during the phases of the pandemic through a longitudinal study.

## METHODS

### Study sample and data collection

This longitudinal study was based on an internet-based survey. The details of this study are only briefly addressed here, since the subject extraction method was described in more detail in our previous study.^13^ In the early phase of the COVID-19 outbreak in Japan, a baseline survey was conducted during February 25–27, 2020. The participants were recruited from MyVoice Communication, Inc., a Japanese Internet research service company with approximately 1.12 million registered participants as of January 2020. Its aim was to collect data from 2,400 men and women aged 20–79 years (sampled by sex and 10-year age groups; *n* = 200 in each of the 12 groups), who were living near the Tokyo metropolitan area across seven prefectures (ie, Tokyo, Kanagawa, Saitama, Chiba, Ibaraki, Tochigi, and Gunma). As of January 2019, the Tokyo metropolitan area, with a total area of 32,433.4 km^2^, is home to approximately 35% of Japan’s total population of 43,512,238. The company invited registrants to participate in the survey by email on February 25, 2020 (*n* = 8,156). The questionnaires were placed in a secured area of a website, and potential respondents received a specific URL in their invitation email. Once 200 participants in each group had voluntarily responded to the questionnaire, the company stopped accepting responses from that group, and after collecting 200 responses from all groups, the survey was concluded on February 27.

**Figure 1.**
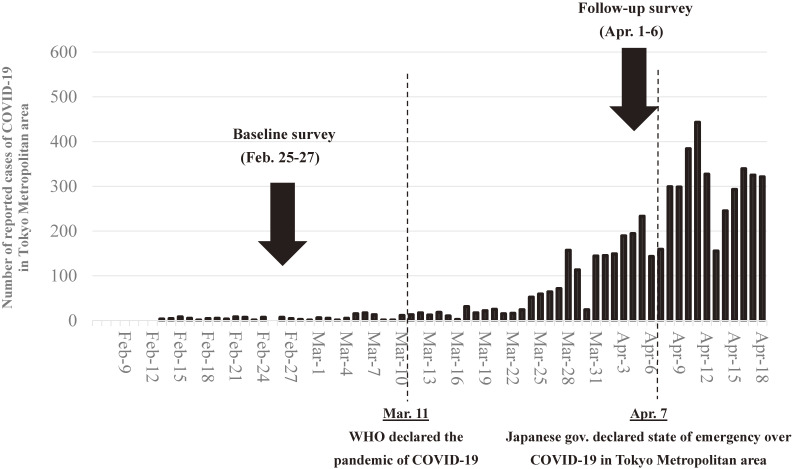
Date of surveys with COVID-19 epidemic curve in Tokyo Metropolitan area

On April 1, Japan reported 2,178 COVID-19 cases, representing a rapid increase in the number of patients, mainly in Tokyo^1^ On the same day, 2,400 respondents from the baseline survey were sent an invitation email to participate in a follow-up survey. The questionnaires were placed in a secured section of a website, and the potential respondents received a specific URL in their invitation email. All 2,400 baseline survey respondents voluntarily responded to the questionnaire, and the cutoff date for completion of the survey was April 6. On April 7, the Japanese government declared a state of emergency.^14^ This study used the data of participants who answered both the baseline and follow-up surveys (*n* = 2,078) (Figure 1).

As an incentive, participants of both the baseline and follow-up surveys were allotted reward points valued at 50 Japanese yen (JPY) (approximately 0.5 United States dollars [USD] based on the prevailing exchange rate in April 2020).

### Measurement

#### Assessment of severe psychological distress

In both the baseline and follow-up surveys, the Kessler’s Six-scale Psychological Distress Scale (K6) was used to measure severe psychological distress (SPD).^15^ The K6 is broadly used in epidemiological studies to assess depression or suicide prevention,^16^^–^^19^ since it measures psychological distress in the general population using six simple items. Each item measures the extent of general nonspecific psychological distress using a 5-point response: 0 “*none of the time*,” 1 “*a little of the time*,” 2 “*some of the time*,” 3 “*most of the time*,” and 4 “*all of the time*”; thus, the total scores ranged from 0 to 24. The K6 was translated into Japanese, and a previous study of 164 Japanese adults showed its internal consistency in relation to reliability (Cronbach’s alpha: 0.849) and validity (100% sensitivity and 69.3% specificity for screening mood and anxiety disorder).^20^ This study used an established protocol to define a score of 13 or above to indicate SPD.^21^

#### Assessment of sociodemographic factors

In the baseline survey, participants reported their sex, age, residential area (Northern Kanto area [Ibaraki, Tochigi, and Gumma Prefectures], Saitama Prefecture, Chiba Prefecture, Kanagawa Prefecture, Tokyo Metropolis), working status (working, not working); marital status (single, divorced, separated, married), living arrangements (alone, with others but without children, with children aged 18 years or older, with others and children under 18 years), annual personal income (less than 2 million JPY [approximately 18,600 USD], 2–<4 million JPY [18,600–<37,200 USD], 4–6 million JPY [37,200–<55,800 USD], 6 million JPY or more [≥55,800 USD]); smoking (smokers, ex-smokers, non-smokers), alcohol consumption (never, seldom [1–4 times/week], often [5–7 times/week]), daily walking time (less than 30 mins, 30–59 mins, ≥60 mins), regular annual vaccination (yes, no), and past medical history (hypertension, diabetes, heart disease, stroke, respiratory disease, kidney disease, cancer). In addition, the research company provided categorized data on educational attainment (junior or high school graduate, junior college graduate, university graduate or above, others).

### Statistical analysis

In the baseline and follow-up surveys, the K6 score was calculated and the *t*-test was used to determine the difference between the two time points among each individual factors. In addition, McNemar’s test was used to examine the percentage of people who scored 13 or more in the K6. To assess the associated factors for changing SPD status between baseline and follow-up surveys, mixed-effects ordinal logistic regression analyses were performed by nesting each participant.^22^ In this analysis, fixed effects for all individual factors were estimated after adjusting total K6 score at baseline. All variables were placed in the model at the same time. All analyses were performed using Stata software version 15 (Stata Corporation, College Station, TX, USA).

### Ethical approval

This study was approved by the Ethics Committee of Tokyo Medical University, Tokyo, Japan (No: T2019-0234). Informed consent was obtained from all respondents.

## RESULTS

Table 1 shows the sociodemographic characteristics of the participants and their SPD percentages during the baseline and follow-up surveys. Of the 2,078 participants, 1,029 (49.3%) were men and the average age was 50.3 (standard deviation [SD], 15.3) years. Approximately 37.2% were workers, of whom 19.1% were living alone. The majority were university graduates or had higher educational attainment. The average K6 scores in the baseline and follow-up surveys were 4.79 (SD, 5.3 points) and 5.60 (SD, 5.4 points), respectively, indicating a significant increase (*P* < 0.001). The percentage of SPD (K6 ≥13) was 9.34% and 11.31% in the baseline and follow-up surveys, respectively, indicating a 2% increase (*P* = 0.005).

**Table 1.** Differences in psychological distress by individual factor

	*n*	%	K6 score (range: 0–24)					Proportion of severe psychological distress (K6 score ≥13)				
	
			Baseline survey (February 25–27, 2020)		Follow-up survey (April 1–7, 2020)		*P*^a^	Baseline survey (February 25–27, 2020)		Follow-up survey (April 1–7, 2020)		*P*^b^
	
			mean	SD	mean	SD		*n*	(%)	*n*	(%)	
Overall	2,078		4.79	5.30	5.60	5.44	**<0.001**	194	9.34%	235	11.31%	**0.005**

Sex												
Male	1,024	49.3%	4.68	5.33	5.45	5.60	**<0.001**	97	9.47%	113	11.04%	0.120
Female	1,054	50.7%	4.90	5.28	5.74	5.27	**<0.001**	97	9.20%	122	11.57%	**0.016**
Age												
20–29 years	288	13.9%	6.62	6.28	7.24	6.46	**0.049**	49	17.01%	56	19.44%	0.297
30–39 years	358	17.2%	6.42	6.41	7.16	6.16	**0.011**	61	17.04%	60	16.76%	0.895
40–49 years	366	17.6%	5.36	5.24	6.19	5.49	**<0.001**	37	10.11%	51	13.93%	**0.048**
50–59 years	356	17.1%	4.25	4.77	5.08	4.89	**<0.001**	25	7.02%	30	8.43%	0.336
60–69 years	363	17.5%	3.43	3.98	4.22	4.38	**<0.001**	13	3.58%	23	6.34%	0.499
70–79 years	347	16.7%	2.97	3.66	3.95	4.15	**<0.001**	9	2.59%	15	4.32%	0.058
Residential area												
Northern Kanto (Ibaraki, Tochigi, Gumma Prefectures)	189	9.1%	5.01	5.43	5.98	5.57	**<0.001**	20	10.58%	23	12.17%	0.439
Saitama Prefecture	336	16.2%	4.90	5.52	5.71	5.88	**0.001**	37	11.01%	43	12.80%	0.317
Chiba Prefecture	300	14.4%	4.44	5.05	5.04	5.04	**0.019**	25	8.33%	26	8.67%	0.862
Tokyo Metropolis	801	38.5%	4.89	5.30	5.70	5.40	**<0.001**	68	8.49%	95	11.86%	**0.003**
Kanagawa Prefecture	452	21.8%	4.69	5.27	5.53	5.36	**<0.001**	44	9.73%	48	10.62%	0.555
Working status												
No	773	37.2%	4.48	5.14	5.39	5.35	**<0.001**	64	8.28%	83	10.74%	**0.012**
Yes	1,305	62.8%	4.98	5.39	5.72	5.49	**<0.001**	130	9.96%	152	11.65%	0.080
Marital status												
Single, divorced, separated	869	41.8%	5.87	5.91	6.48	6.14	**<0.001**	125	14.38%	141	16.23%	0.127
Married	1,209	58.2%	4.02	4.67	4.96	4.78	**<0.001**	69	5.71%	94	7.78%	**0.015**
Living arrangement												
Living alone	396	19.1%	5.14	5.42	5.81	5.68	**0.005**	45	11.36%	52	13.13%	0.336
Living with others but without children	991	47.7%	4.99	5.52	5.80	5.72	**<0.001**	100	10.09%	128	12.92%	**<0.001**
Living with children aged ≥18 years	349	16.8%	3.39	3.86	4.36	4.29	**<0.001**	12	3.44%	19	5.44%	0.127
Living with children aged <18 years	342	16.5%	5.25	5.56	6.01	5.19	**0.005**	37	10.82%	36	10.53%	0.879
Education (years)												
Junior or high school graduate (≤12 years)	490	23.6%	5.16	5.56	6.12	5.88	**<0.001**	56	11.43%	68	13.88%	0.101
Junior college graduate (13–15 years)	441	21.2%	4.67	4.75	5.56	5.09	**<0.001**	33	7.48%	43	9.75%	0.114
University graduate or above (≥16 years)	1,122	54.0%	4.64	5.34	5.38	5.36	**<0.001**	101	9.00%	122	10.87%	**0.050**
Other	25	1.2%	6.56	6.92	5.40	5.73	**0.383**	4	16.00%	2	8.00%	0.317
Smoking status												
Smoker	311	15.0%	4.81	5.30	5.70	5.50	**<0.001**	29	9.32%	37	11.90%	0.206
Ex-smoker	303	14.6%	4.21	4.97	4.77	5.05	**0.039**	23	7.59%	29	9.57%	0.273
Non-smoker	1,464	70.5%	4.91	5.36	5.74	5.49	**<0.001**	142	9.70%	169	11.54%	**0.025**
Alcohol consumption												
None	882	42.4%	5.10	5.70	5.78	5.62	**<0.001**	103	11.68%	105	11.90%	0.838
Seldom (1–4 days/week)	741	35.7%	4.74	5.05	5.71	5.31	**<0.001**	62	8.37%	84	11.34%	**0.009**
Often (5–7 days/week)	455	21.9%	4.27	4.85	5.06	5.25	**<0.001**	29	6.37%	46	10.11%	**0.015**
Walking time, mins/day												
<30	1,047	50.4%	5.10	5.51	5.89	5.63	**<0.001**	116	11.08%	138	13.18%	**0.039**
30–59	687	33.1%	4.39	4.88	5.33	5.18	**<0.001**	50	7.28%	66	9.61%	0.052
≥60	344	16.6%	4.66	5.41	5.23	5.30	**0.034**	28	8.14%	31	9.01%	0.602
Regular vaccinations												
Yes	1,159	55.8%	4.85	5.50	5.48	5.54	**<0.001**	119	10.27%	136	11.73%	0.119
No	919	44.2%	4.72	5.04	5.74	5.31	**<0.001**	75	8.16%	99	10.77%	**0.014**
Annual personal income, United States dollars												
<18,600	936	45.0%	6.03	6.03	6.22	5.72	**<0.001**	106	11.32%	129	13.78%	**0.028**
18,600–<37,200	531	25.6%	4.61	5.06	5.25	5.41	**0.004**	54	10.17%	60	11.30%	0.453
37,200–<55,800	312	15.0%	4.24	4.86	5.44	5.01	**<0.001**	22	7.05%	26	8.33%	0.479
≥55,800	299	14.4%	4.15	4.90	4.43	4.72	**0.006**	12	4.01%	20	6.69%	0.074
Comorbidities												
Hypertension	395	19.0%	4.24	4.79	4.94	4.86	**<0.001**	26	6.58%	33	8.35%	0.178
Diabetes	123	5.9%	4.55	4.88	4.53	5.23	0.936	10	8.13%	12	9.76%	0.480
Heart disease	62	3.0%	4.89	5.02	6.42	5.76	**0.003**	5	8.06%	10	16.13%	0.059
Stroke	20	1.0%	6.00	7.18	6.25	7.27	0.536	4	20.00%	4	20.00%	1.000
Respiratory disease	89	4.3%	6.36	5.68	7.67	6.30	**0.006**	16	17.98%	22	24.72%	0.157
Kidney disease	10	0.5%	7.30	7.18	8.20	7.13	0.430	1	10.00%	2	20.00%	0.317
Cancer	43	2.1%	5.33	5.35	5.86	5.13	0.477	2	4.65%	3	6.98%	0.564

K6, Kessler’s Six-scale Psychological Distress Scale; SD, standard deviation.

^a^*P*-value was calculated using paired *t*-test.

^b^*P*-value was calculated using McNemar’s test.

Bold values denote statistical significance at *P* < 0.05.

Table 2 shows the results of a mixed-effects ordinal logistic regression analysis. Compared to those with higher income (ie, ≥55,800 USD of annual personal income), significantly high likelihood to develop SPD were observed among those in lower (ie, 18,600–37,200 USD, odds ratio [OR] 1.95; 95% confidence interval [CI], 1.10–3.46) and the lowest income category (ie, <18,600 USD, OR 2.12; 95% CI, 1.16–3.86). Furthermore, those with respiratory diseases were more likely to develop SPD (OR 2.56; 95% CI, 1.51–4.34).

**Table 2.** Individual factors associated with development of severe psychological distress: mixed-effect ordinal logistic regression results

	OR^a^	95% CI	*P*
Gender			
Male	1.00		
Female	0.87	(0.63–1.20)	0.389
Age			
20–29 years	1.26	(0.73–2.16)	0.403
30–39 years	1.22	(0.73–2.05)	0.449
40–49 years	1.39	(0.84–2.32)	0.202
50–59 years	1.00		
60–69 years	1.04	(0.59–1.83)	0.899
70–79 years	0.79	(0.40–1.56)	0.497
Residential area			
Northern Kanto (Ibaraki, Tochigi, or Gumma Prefectures)	1.01	(0.62–1.65)	0.963
Saitama Prefecture	1.22	(0.82–1.82)	0.321
Chiba Prefecture	1.02	(0.65–1.59)	0.942
Tokyo Metropolitan	1.00		
Kanagawa Prefecture	1.13	(0.79–1.63)	0.507
Working status			
No	1.11	(0.77–1.59)	0.590
Yes	1.00		
Marital status			
Never married, divorced, or separated	1.06	(0.71–1.60)	0.767
Married	1.00		
Living arrangement			
Living alone	1.08	(0.74–1.60)	0.682
Living with others but without children	1.00		
Living with children aged ≥18 years	0.94	(0.55–1.59)	0.811
Living with children aged <18 years	0.85	(0.52–1.36)	0.491
Education (years)			
Junior or high school (≤12 years)	0.98	(0.69–1.39)	0.914
College (13–15 years)	0.99	(0.68–1.45)	0.967
University or higher (≥16 years)	1.00		
Others	0.29	(0.07–1.26)	0.098
Smoking status			
Current	1.00		
Quit	1.17	(0.78–1.74)	0.446
Never	1.21	(0.78–1.87)	0.388
Drinking alcohol			
No	1.00		
Seldom (1–4 days/week)	1.11	(0.81–1.52)	0.517
Often (5–7 days/week)	1.16	(0.78–1.74)	0.458
Walking time, min/day			
<30	1.47	(0.96–2.24)	0.075
30–59	1.27	(0.80–2.00)	0.306
≥60	1.00		
Vaccinated regularly			
Yes	1.00		
No	1.09	(0.68–1.73)	0.724
Annual personal income, United States dollars			
<18,600	**2.12**	**(1.16–3.86)**	**0.014**
18,600–<37,200	**1.95**	**(1.10–3.46)**	**0.022**
37,200–<55,800	1.19	(0.65–2.17)	0.572
≥55,800	1.00		
Comorbidities			
Hypertension	0.82	(0.53–1.27)	0.374
Diabetes	1.27	(0.67–2.43)	0.460
Heart disease	1.71	(0.74–3.97)	0.210
Stroke	1.30	(0.26–6.50)	0.746
Respiratory disease	**2.56**	**(1.51–4.34)**	**<0.001**
Kidney disease	0.64	(0.08–5.03)	0.668
Cancer	0.34	(0.09–1.31)	0.117

Baseline K6 score	**1.43**	**(1.39–1.48)**	**<0.001**

K6, Kessler’s Six-scale Psychological Distress Scale; OR, odds ratio.

Bold values denote statistical significance at the *P* < 0.05 level.

^a^Odds ratios were calculated with adjustment for all other variables (ie, gender, age, residential area, working status, marital status, living arrangement, education, smoking status, drinking alcohol, walking time, regular vaccination, annual personal income, comorbidities and K6 score at baseline).

## DISCUSSION

### Summary of findings

We set out to determine the degree of change in the psychological distress of the general population in the Kanto region between the early and transmission phases of COVID-19, and the characteristics of those who displayed a significant change. The results demonstrated that the mental health of the general population had significantly deteriorated from the early phase to transmission phase. The degree of deterioration was more remarkable among those with respiratory diseases and those with low incomes.

### Overall impact

This study was able to confirm the degree of deterioration and determine causal factors. In an interview survey conducted in the United Kingdom on the general population and psychiatric patients, the causes for the deterioration of mental health were identified as: i) anxiety caused by uncertainty, ii) increased sense of isolation due to social distancing policy, iii) diminished medical access, and iv) family relations (eg, family concerns, domestic violence).^23^ In fact, there is evidence that suicide deaths increased due to the 1918–19 influenza pandemic.^24^ Therefore, it can be suggested that mental health measures should be implemented together with other protective measures against COVID-19 infection.

### High-risk groups

In this study, a high degree of deterioration was observed among low-income individuals, which we believe may have been affected by a decrease in income between the two phases. On March 28, 2020, the Japanese government introduced the “Basic Policy for Novel Coronavirus Disease Control”.^25^ This policy strongly urged the public to refrain from going outside unless it was absolutely necessary, to reduce social interaction, and to work remotely as much as possible. This also included the suspension of services involving the congregation of people; therefore, various businesses, such as fitness facilities, restaurants, and concert venues, closed temporarily. Speculatively, a majority of those who work at such facilities are part-time or temporary workers, most often individuals with low incomes. It is possible that the suspension of these businesses may have greatly reduced their income or even led to their dismissal, thus posing a threat to their daily lives.

In the past, the number of suicides increased in central Hong Kong due to the economic impact during the 2003 outbreak of severe acute respiratory syndrome (SARS).^26^ Similarly, there was a concern that suicide cases might increase during the COVID-19 pandemic for various reasons, including economic loss.^6^ It is still uncertain what the future holds for Japan. Many countries are providing financial support to cover the loss of income due to the pandemic. As this study has shown a deterioration in mental health earlier than others, it may be important to provide such financial support at an early stage for low socio-economic status groups.

### Impact on people with underlying diseases

It has been pointed out that the mental health of those with underlying diseases might further deteriorate.^5^ This study revealed that mental health worsened in people with respiratory diseases, among other underlying diseases. This may have been caused by the fear of the possibility of becoming severely ill as a result of infection. Another reason may be that during the shift from the early phase to the transmission phase, medical facilities had no choice but to concentrate on coronavirus treatment, causing limited access for these patients. Providing support, such as by expanding online medical consultations, for those with respiratory diseases may be necessary to enable patients to continue treatment without anxiety.

### Strengths and limitations

There are some limitations to our study that should be considered. First, selection bias in the web-based internet survey could have been introduced. According to a 2019 white paper, regular internet-users were younger age and had higher income compared to non-users.^27^ Older adults in the present study may have a higher income than average. In addition, loss to follow-up occurred more frequently among youth, never smokers, those who live with children aged >18 years, and those who do not take vaccines regularly (data not shown), which may cause selection bias. Second, the results may not be directly applicable to the Japanese population due to limited representativeness. Age- and gender-stratified sampling causes different distributions of individual characteristics, compared to Japanese population. In addition, the study participants were recruited from the Tokyo metropolitan area only. Furthermore, the level of psychological distress among younger age-groups was higher than the national average.^28^ Taken together, future research would be needed to investigate the change of psychological distress, especially among youth in non-Tokyo areas. Third, no data on current or past history of medication for mental health were obtained for this study. If a certain number of participants started medication during the period of the two surveys, the results may be biased. Finally, the sample size is not sufficiently large; hence, this study may overlook the true association due to lower statistical power. For example, those with heart disease or kidney disease showed no significant association, despite the high point estimates. Future studies with larger sample sizes would be preferable.

### Conclusion

From the early to the community-transmission phases of COVID-19, mental health among Japanese people deteriorated. Therefore, it can be suggested that mental health measures be implemented together with protective measures against COVID-19 infection. In particular, high priority should be given to low-income people and those with underlying diseases, who may be prone to deterioration of mental health.
